# Simultaneous label-free live imaging of cell nucleus and luminescent nanodiamonds

**DOI:** 10.1038/s41598-020-66593-7

**Published:** 2020-06-17

**Authors:** Michal Gulka, Hamideh Salehi, Bela Varga, Elodie Middendorp, Orsolya Pall, Helena Raabova, Thierry Cloitre, Frederic J. G. Cuisinier, Petr Cigler, Milos Nesladek, Csilla Gergely

**Affiliations:** 1 Institute for Materials Research (IMO), Hasselt University, Wetenschapspark 1, B-3590 Diepenbeek, Belgium; 2 Department of Biomedical Technology, Faculty of Biomedical Engineering, Czech Technical University in Prague, Sítná sq. 3105, 272 01 Kladno, Czech Republic; 3 Laboratoire de Bioingénierie et Nanoscience (LBN), Université de Montpellier, Montpellier, France; 4 Laboratoire Charles Coulomb (L2C), Université de Montpellier, CNRS, Montpellier, France; 5 Institute of Organic Chemistry and Biochemistry of the CAS, Flemingovo nam. 2, 166 10 Prague 6, Czech Republic

**Keywords:** Nanoscale materials, Microscopy

## Abstract

In recent years, fluorescent nanodiamond (fND) particles containing nitrogen-vacancy (NV) centers gained recognition as an attractive probe for nanoscale cellular imaging and quantum sensing. For these applications, precise localization of fNDs inside of a living cell is essential. Here we propose such a method by simultaneous detection of the signal from the NV centers and the spectroscopic Raman signal from the cells to visualize the nucleus of living cells. However, we show that the commonly used Raman cell signal from the fingerprint region is not suitable for organelle imaging in this case. Therefore, we develop a method for nucleus visualization exploiting the region-specific shape of C-H stretching mode and further use *k*-means cluster analysis to chemically distinguish the vicinity of fNDs. Our technique enables, within a single scan, to detect fNDs, distinguish by chemical localization whether they have been internalized into cell and simultaneously visualize cell nucleus without any labeling or cell-fixation. We show for the first time spectral colocalization of unmodified high-pressure high-temperature fND probes with the cell nucleus. Our methodology can be, in principle, extended to any red- and near-infrared-luminescent cell-probes and is fully compatible with quantum sensing measurements in living cells.

## Introduction

Thanks to convenient non-bleaching luminescence and room-temperature magnetic properties, fluorescent nanodiamonds (fNDs) are highly attractive as ultrasensitive nanoscale quantum sensors that can be used for intracellular imaging and sensing, exploiting both photoluminescence (PL)^1–4^ and magnetic resonance detection of the NV centers^5–8^. Recent technological developments facilitated fabrication^9–11^ of bright fNDs of various sizes and with various content of nitrogen-vacancy (NV) centers^12^, allowing thus to optimize the sensing performance. Since the demonstration of NV spin readout inside living cells in 2011^6^, important progress has been made in utilizing the properties of NV spins at ambient conditions for various applications ranging from relaxometry sensing using T_1_ sequence^7,13,14^ to local cell temperature measurements^15,16^. Yet much more is expected thanks to NV exceptional sensitivity and the non-disturbing character of the detection mechanism.

The NV sensing techniques with fNDs open possibilities to study biological processes at a quantum level with unprecedented spatial resolution. However, further information would be gained, if the NV detection were combined with chemical information of the nearest cellular environment. Such a combination of quantum sensing experiments with simultaneous localization of fNDs with respect to organelles, however, remains a challenge. Determination of fNDs location in cell with respect to the nucleus of a non-fixed non-labeled cell is also crucial for many other applications. These include fND-mediated drug^17^ and gene delivery^18^ or studies of cellular transport (e.g. of proteins^19^). Current methods for nucleus visualization employ either color staining with additional fluorescent dyes^20^ and require fixation of cells^21^. These procedures, however, alter the natural state of the cells or even interfere with nuclear function^22^. In addition, dye bleaching prevents long-term imaging and the cell-fixation excludes the live time-dependent intracellular measurement. For some cells, the position of the nucleus can be estimated from the confocal image of cells auto-fluorescence. This has been shown on living human cervical cancer (HeLa) cells, where a 3D image of the cell was reconstructed and combined with the image of luminescent nanodiamonds detected with two-photon excitation microscopy^23^. Yet, a technique that could precisely and reproducibly distinguish the cell organelles and at the same time localize luminescent probes (i.e. fNDs and other red/near-infrared luminescent probes, such as quantum dots and carbon nanotubes) within the biochemical environment of the cell would be very valuable. Especially if it could visualize the cell nucleus with high contrast and would be compatible with NV-based intracellular quantum imaging and sensing^24^.

**Figure 1:**
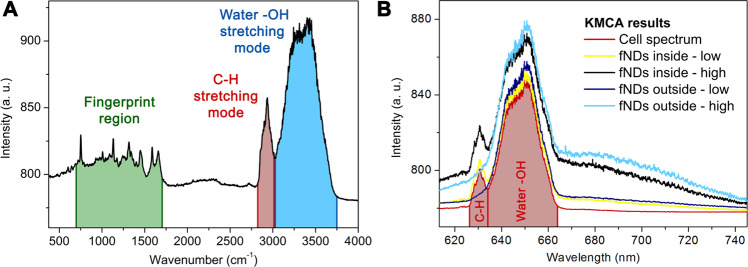
PL/Raman measurements demonstrated in living MCF7 cells. (**A**) Typical spectra obtained from non-incubated cells with the central wavelength of grating set at 610 nm (the common setting for Raman cell imaging) showing the “fingerprint region” from ~700 to ~1700 cm^−1^, the C-H peak at 2915 cm^−1^ from carbohydrates within the cell and H_2_O peak at 3400 cm^−1^ from the DPBS solution used for measurement. (**B**) The average spectral shape of KMCA clusters obtained after cell incubation with fNDs. The central wavelength of grating is shifted to 680 nm. In total five clusters were created with KMCA. The cluster corresponding to the cell signal (non-NV-luminescent) is shown in red and four NV-luminescent clusters are shown in light blue, dark blue, yellow, and black (see methods). With the central wavelength of grating set at 680 nm we increase the fNDs detection sensitivity more than six times (see Supplementary Information), however, we lose the “fingerprint region” as a consequence.

One of the techniques known for chemical analysis of the cellular environment is Raman spectroscopy and imaging^25^. Raman imaging is a well-established powerful tool to visualize organelles of living cells by analyzing the Raman pixel spectra^26–30^. The most straightforward option to identify different cell compartments is to analyze the so-called “fingerprint region”^31^. This region lays from ~700 to ~1700 cm^−1^ and is the most important segment of the cell spectrum as it contains information of specific cell parts. It has been shown^28^ that by *k*-means cluster analysis (KMCA) this information can be processed to differentiate between various cell organelles (including the nucleus^32^, nucleolus, mitochondria or cytoplasm), as each organelle exhibits different intensity of specific Raman peaks. The “fingerprint region” can be detected for example using 532 nm excitation wavelength, which is the same color standardly used for NV excitation and spin readout. However, the common spectral detection window for “fingerprint” detection (see Fig. 1A) is ranging roughly from 100 to 4000 cm^−1^ ^27^, which corresponds to ~535 and ~675 nm respectively, whereas almost 70% of the NV emission lies in the 670 − 890 nm region^33^. Even though some of the NV luminescence would be still detected (NV centers exhibit luminescence with zero-phonon lines [ZPL] of 575 nm for NV° and 636 nm for NV^−^ charge states, but only about 4% of NV photons are emitted into the ZPL^34^), this approach (see Supplementary Information) is impractical and yields only low recognition of fND particles due to the spectral overlap of Raman signals from cell and water with the NV luminescence. It is also important to emphasize that for purposes of live-cell imaging and quantum sensing it is rather imperative to obtain the image in one scan due to the sample drift and defocusing during the measurements.

Other Raman-based methods are stimulated Raman scattering (SRS), coherent anti-Stokes Raman scattering (CARS), or surface-enhanced Raman scattering (SERS) microscopy. However, none of these techniques is compatible with pulsed protocols used for NV sensing and/or simultaneous single scan visualization of luminescent NDs and label-free live-cell imaging of the nucleus. Both SRS and CARS make use of fast pulsed laser sources (SRS ps to fs^35^, CARS ps range^36^) with wavelength higher than 800 nm to reduce multiphoton absorption^36^, whereas green laser is necessary to maximize the PL detection of fNDs. SERS, on the other hand, relies on DNA intercalation staining to visualize the nucleus^37^. It is thus not a label-free method and is used to detect noble metallic nanostructures. Moreover, for all of these Raman techniques, the detection window again does not match the region of high NV luminescence intensity. We have also considered other nonlinear optical microscopy techniques, such as two-photon excitation fluorescence, second- and third-harmonic generation, but none of these methods meet the requirements for single scan label-free detection of the cell nucleus and fNDs (due to the spectral overlap).

Here we present a combination of the Raman imaging method with sensitive localization of fNDs, which allows NV luminescence detection merged with confocal Raman microscopy of the cellular environment in a single scan. This methodology utilizes the C-H Raman peak for nucleus visualization, which enables to shift the spectral detection window to longer wavelengths and collect the majority of the NV photons, thus achieving a sensitive chemical localization of even very small (5–50 nm) fNDs. Our approach employs the C-H stretching mode, which is a vibrational mode of the C-H groups and one of the most intense Raman mode found in cells. It is abundant in proteins, lipids, and carbohydrates, and an image based on the intensity of this band provides a pseudo-map of protein, lipid, and carbohydrate concentration in cells^38^. Therefore, the chemical contrast of the nucleus can be based on the protein/lipid ratio in the cell compartments, which is expressed by the shape of the C-H stretching mode. By mapping a specific part of the C-H stretching mode that primarily corresponds e.g. to the lipid signal, we show that the nucleus can be visualized clearly. Unlike the KMCA of the “fingerprint region”, where specific peaks are sorted by the shape and intensities, here we simply create an intensity map of a specific spectral region. A similar technique to identify cell nucleus has been proposed in coherent anti-Stokes Raman scattering (CARS) measurements^39,40^ by tuning the beating frequency to 2845 cm^−1^ to probe the CARS signals originating from lipid part of symmetric CH_2_ stretching bonds^36^. In CARS, the optical parametric oscillator is necessary to obtain the signal.

In our approach, the visualization of the nucleus with the C-H peak is performed using a standard commercial Raman microscope with a continuous wave laser and in combination with KMCA-detected localization of fNDs from the NV luminescence in the pixel spectrum (see Materials and Methods). Our method is thus compatible with NV quantum sensing schemes, such as quantum NMR detection and imaging^5^. Thanks to this technique, we demonstrate the colocalization of the luminescent probes with cell organelles by identifying the fNDs in the area of the cell nucleus. We validate our method on mammalian breast cancer (MCF7) cell line, mammalian breast cell line (184A1), and human dental pulp stem cells (DPSC) incubated with luminescent fNDs and we show that this method can be employed to both living and fixed cells. The resolution of our method is diffraction-limited but could be enhanced by super-resolution techniques. Since we detect mainly the PL phonon sideband of NVs, this technique can be in principle applied to any red/near-infrared probes. Within a single scan, we are able to record the characteristic NV spectra, identify the luminescent pixels, distinguish between fNDs outside or inside the cell, readout the C-H Raman stretching region information, visualize the nucleus of unstained living cells with high contrast and by superposing all this information create the resulting image with confocal resolution.

## Materials and Methods

### Sample preparation

High-pressure high-temperature (HPHT) luminescent fND particles of size range from 5 to 50 nm^41^, with the vast majority of particles of 20 nm size (as confirmed by dynamic light scattering and atomic force microscopy), were obtained from Microdiamant, Switzerland (MSY 0–0.05). The purified and oxidized fNDs were irradiated in an external target holder for 12 hours with a 16.6 MeV electron beam (8.11 × 10^18^ particles cm^−2^) from MT-25 microtron^42^. The irradiated material was annealed at 900 °C for 1 h^3^ and oxidized again in the air at 510 °C for 6 h.

### Cell experiments

Cells used for the experiments were breast cancer cells (MCF7), mammalian breast cells (184A1), and human dental pulp stem cells (DPSC). The choice of cells ranges from resilient cancerous cells to more delicate DPSC with a goal to demonstrate our method on various cell types. Cells were grown on CaF_2_ substrate in complete medium (10% FBS + 100 μg/ml penicillin-streptomycin) at 37 °C and 5% CO_2_ for 2–3 days to obtain the desired confluency. MCF7 were grown in Dulbecco’s modified eagle’s medium (DMEM), 184A1 in MEGM and DPSC in αMEM. The fNDs, diluted in distilled deionized water (4 mg/ml), were filtered through a 200 nm pore-size membrane to exclude bacteria or larger clusters of nanoparticles. The resultant concentration of fNDs after filtration was determined by gravimetric analysis and the solution was further diluted in distilled deionized water to obtain an fND concentration of 1 mg/ml. The fNDs solution was gradually added to the fetal bovine serum (FBS) to prevent aggregation of fNDs in cell medium^43^ similarly as described recently^44^. Lastly, this mixture was diluted in the cell medium to obtain a concentration of 30 µg/ml fNDs in medium containing 10% FBS. The fNDs in complete media were heated up to 37 °C prior to incubation and the original cell medium was completely removed and replaced by the fND-containing medium. Incubated cells were kept at 37 °C and 5% CO_2_ for 1 hour. For live-cell imaging, samples were rinsed 5 times in Dulbecco’s Phosphate-Buffered Saline (DPBS) and were kept in DPBS for the Raman measurements. For the cell fixation, 2% of paraformaldehyde for 15 minutes was used at room temperature (RT). The fixed samples were rinsed 3–4 times with DPBS and afterward were kept in DPBS and stored in the fridge at 4 °C until the measurement.

### Raman imaging

The Raman spectra were collected using a Witec’s Confocal Raman Microscope Alpha System 300 R (Witec Inc., Ulm, Germany). The excitation for the confocal Raman microscope was provided by a frequency-doubled Nd:YAG laser (Newport, Evry, France) at the wavelength of 532 nm, with 50 mW laser output power and 20 mW laser power at the output of the objective. The incident laser beam was focused onto the sample through a 60x NIKON water immersion objective with the numerical aperture of NA = 1.0. The signal was captured by an electron-multiplying charge-coupled device (EMCCD) camera (DU 970 N-BV353, Andor, Hartford, USA). The spatial resolution of the system is calculated by the formula r_lateral_ = 1.22·λ_laser_/2·NA, which is 325 nm and the axial resolution is determined by r_axial_ = 1.4·λ_laser_·n/NA^2^ (where n is the index of refraction – 1.33 for the water-based objective) and is 991 nm for our system. Data acquisition and processing were performed using the Image Plus software from Witec.

### Combined photoluminescence/Raman imaging of fNDs in cells

CaF_2_ was used as a cell substrate and its characteristic Raman peak at 320 cm^−1^ was employed as an aide for the focusing. The Raman spectra were recorded in a single scan from each pixel of the image as the confocal PL/Raman system was scanning through the sample. To create an image, we make use of advanced data analysis methodology based on two steps. First, we utilized integrated Raman vibration mode intensities of the C-H stretching mode to create the cell image by mapping the intensities of the C-H peak or by mapping a specific part of the C-H peak to achieve the visualization of the cell nucleus. The second part of the post-processing consists of *k*-mean clusters analysis (KMCA) of the recorded spectra. KMCA is a simple unsupervised algorithm used to solve the clustering problem^45^ and is often utilized in cell biology for spectral image analysis. It is applied to identify specific Raman information in the spectra and to cluster similar image pixels together based on user-defined criteria (such as the presence of the peaks and their relative intensities). The procedure follows a way to classify a given data set through a certain number of clusters (assume *k*-mean clusters) and cycles until the differences between the data in each cluster are minimized. Though commonly used to identify the cell compartments, we include the NV PL into the cluster analysis together with the Raman signal to enable simultaneous detection of fNDs and cell nuclei. First, we assess the relative PL intensity of NVs in the pixel by PL intensity thresholding. Initially, two clusters are created using KMCA – a cluster of pixels without recognizable NV luminescence and pixels where the presence of NV luminescence is obvious. Further, pixels that contain NV luminescence are sorted into two additional clusters depending on whether the signal comes from the cells (by the presence of the C-H peak).

Note, that with KMCA the NV PL signal is visualized differently than in classical confocal luminescence microscopy. Here, every pixel identified as luminescent is highlighted with the same color. Therefore, the pixels that contain signal from a few or many NV centers would look alike in the image. To differentiate between e.g. single fNDs and fND aggregates we divided the luminescent clusters into an additional two clusters depending on the NV luminescence intensity (high or low) and then distinguished them with a different pixel color. This differentiation can be further fine graded if necessary. The fNDs outside the cells were marked dark blue for low-intensity (both in spectra and images) and light blue for high-intensity PL signal (both in spectra and images). The fNDs inside the cells with low-intensity PL signal were marked yellow (both in spectra and images) and those with high-intensity black (spectra) or white (images). See Fig. S1 of Supplementary Information. Care was taken when selecting the threshold for NV luminescence to prevent false positive detection of fNDs.

The demonstrated nucleus visualization by C-H peak analysis, the KMCA, and the creation of dual-image are based on post-processing of data collected in one single scan. Since we do not need to collect the fingerprint region where longer integration times are necessary, we can perform fast scans on a timescale from 1 hour down to few minutes (depending on the scan size and the desired pixel size). Thus, the sample illumination and duration of the scan are the same or lower as for standard Raman or PL imaging of living cells and do not induce phototoxicity, as we demonstrated previously even for longer exposures in the same illumination conditions^28,46^. Additionally, we did not observe any smudging of the NV signal due to the NDs movement in live cells. This was true even for particles trapped in the lysosomes^47^, as the pixel recording speed was more than an order of magnitude faster than the lysosome movement^48^.

## Results and discussions

The standard Raman cell spectrum with the central wavelength of the grating set to 610 nm is displayed in Fig. 1A and shows the so-called “fingerprint region”^31^, C-H stretching mode, and –OH stretching mode. Initially, we imaged the internalized fNDs using the standard grating setting, but it resulted in low recognition of fNDs. By shifting the grating to 680 nm the sensitivity improves dramatically leading to more than six times higher detection rate (see Supplementary Information for more detail). Therefore, we carried out our further experiments with the grating central wavelength set to 680 nm, losing the “fingerprint region” in the process. The images are then created by analyzing the combined PL/Raman signal (see Materials and Methods) and consist of two stacked layers. The first layer is the cell image created by mapping the intensity of the C-H stretching mode. On top, we highlight the pixels that contain the NV luminescence in their spectrum employing the KMCA. A typical set of average spectra resulted from KMCA-sorted clusters are shown in Fig. 1B.

### C-H Raman nucleus chemical imaging

**Figure 2:**
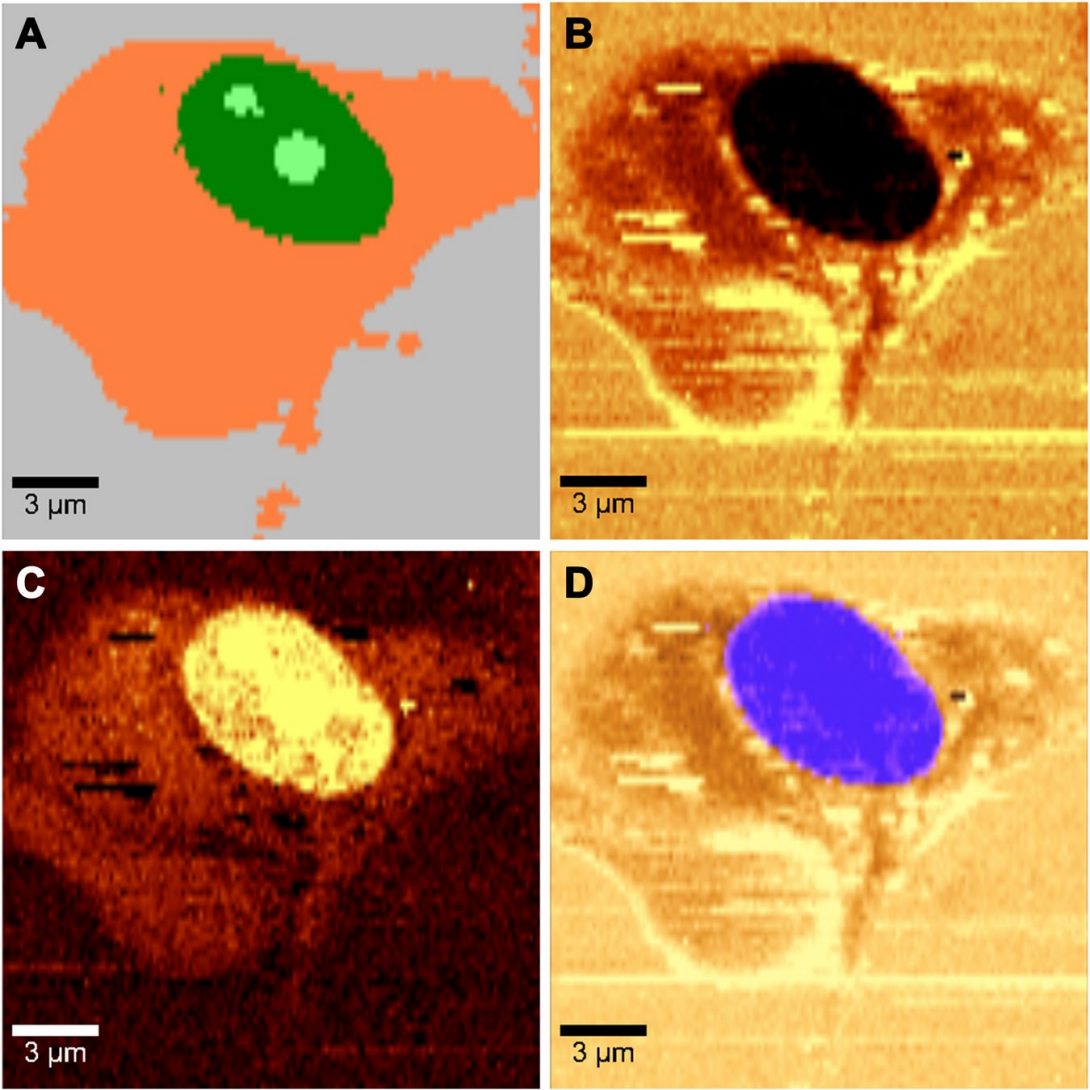
Comparison of nucleus visualization using KMCA of “fingerprint region” and mapping of C-H peak intensities in specific regions of non-incubated living MCF7 cells. All four pictures come from the same measurement; we only employ different spectral analysis. Figure (**A**) shows nucleus visualization using the classic approach of KMCA of “fingerprint region” (cell is shown in orange, the nucleus in dark green and nucleolus in light green), (**B**) using “negative image” obtained by mapping the 2800–2935 cm^−1^ C-H region, (**C**) using “positive image” obtained by mapping the 2930–3010 cm^−1^ C-H region and figure (**D**) shows an agreement between the KMCA and “negative image” methods by merging the image B (in brown) with the nucleus and nucleolus clusters from image A (in blue).

Since we have lost the cell signal from the “fingerprint region”, we exploited the C-H Raman stretching mode for sensitive one-scan detection of fNDs together with chemical analysis of the cellular environment. The Raman signal of C-H stretching mode comes mainly from proteins, lipids, and carbohydrates within the cells^31^ and the exact shape and position of this peak differ for different concentrations of these molecules and their ratios in the detected confocal voxel volume. It has been shown that the C-H peak consisting of the protein signal (or similarly of the compact DNA signal^49^) is right-shifted to higher wavenumbers corresponding to 2920 cm^−1^ whereas the lipid signal is left-shifted to lower wavenumbers corresponding to 2855 cm^−1^ ^50^. Since there is a predominance of compact DNA in the nucleus^51^ and there is only a very low concentration of lipids^52,53^, this leads us to the idea to map only a specific part of the C-H peak, i.e. either corresponding to the protein or the lipid signal and thus create contrast between the nucleus and rest of the cell based on the difference in protein/lipid ratio. To validate this methodology, we imaged living MCF7 cells using standard grating settings (central wavelength at 610 nm) and compared the commonly used “fingerprint region” method with our method using the C-H peak. In Fig. 2A the nucleus is visualized using the standard KMCA of the “fingerprint region” following the procedure described in^27^. In Fig. 2B, using the same data set, we mapped the intensities of the C-H band in the range of 2800–2935 cm^−1^ to highlight the areas with a predominance of lipid signal. It can be clearly seen that this method creates a very high contrast of the nucleus shown in dark compared to the rest of the cell (therefore, we call it “negative image”). This distinction comes from the lack of lipids in the cell nucleus in comparison to the other organelles. An alternative approach is to map C-H intensities in the range of 2930–3010 cm^−1^ to highlight the areas with a predominance of protein signal. The resulting image is shown in Fig. 2C. Here the nucleus is brighter than the rest of the cell (therefore we call it “positive image”) because the protein/lipid ratio is higher in the nucleus. Yet, as the protein signal is relatively high everywhere within the cell, the contrast is lower compared to “negative image”. Nevertheless, the nucleus can be still recognized clearly. To compare the “negative imaging” technique of C-H peak with common KMCA nucleus visualization we merge the images obtained by these methods (Fig. 2D). We get an excellent agreement of the nucleus position indicated by a perfect overlap of the nucleus images.

### Simultaneous fNDs and nucleus imaging

**Figure 3:**
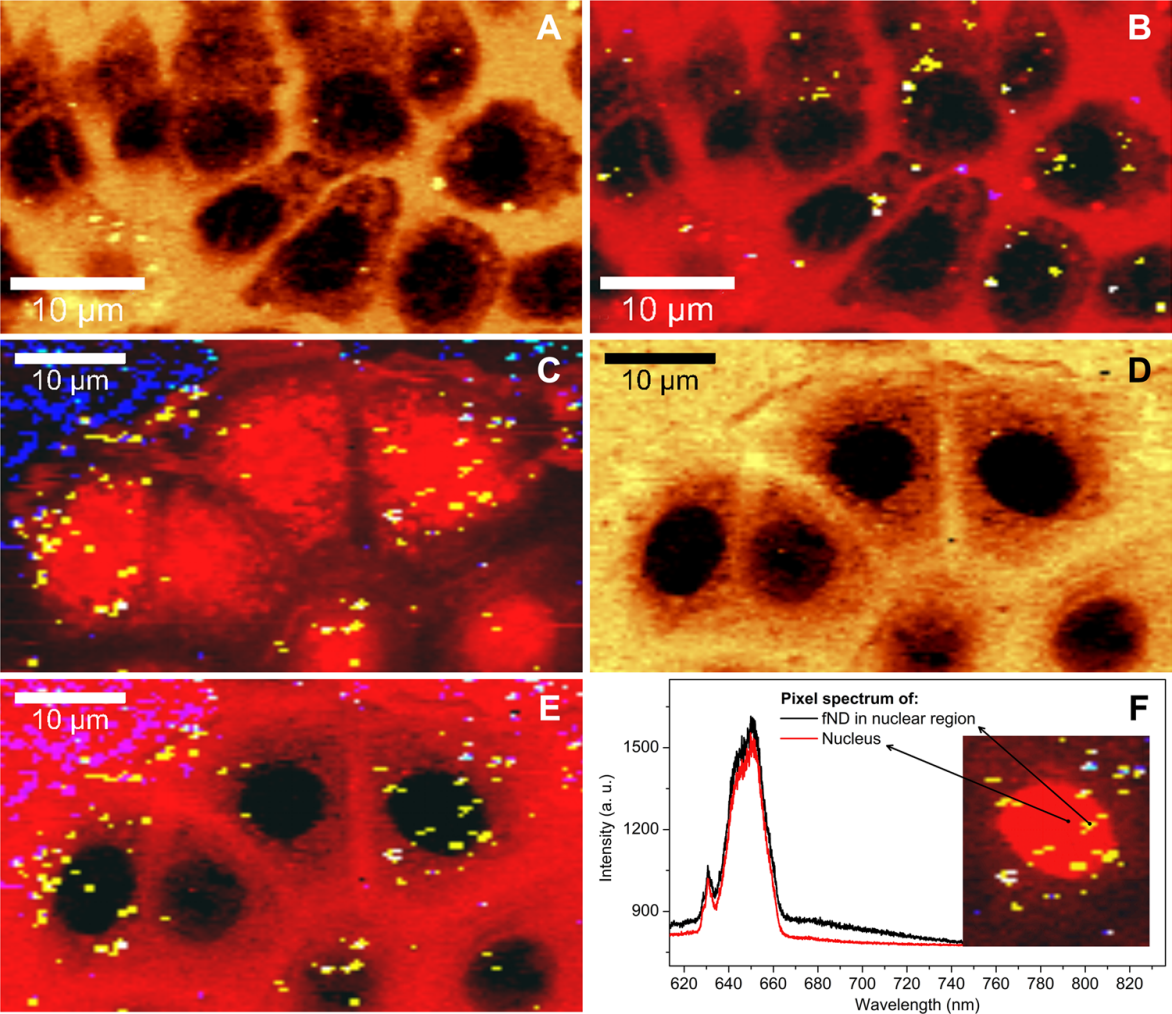
Demonstration of PL/Raman visualization of living (**A**,**B**) and fixed (**C**,**D**,**E**,**F**) MCF7 cells by mapping the specific range or full-range of the C-H peak and simultaneous detection of NV-luminescent pixels using KMCA. The image (**A**) shows the visualization of the cell nucleus (note that the yellow spots in the image are lipids aggregations and not fNDs) and in (**B**) this information is combined with KMCA detected nanodiamonds. Image (**C**) is created using standard full-range C-H intensity mapping with KMCA detected fNDs. Image (**D**) shows the “negative image” of the cells using our method and in (**E**) this image is combined with detected fNDs. The combination of the luminescence detection and spectral information from cell resulted in the colocalization of the fNDs with the cell nucleus. The individual pixel spectra from the nucleus with and without NV luminescence are shown in (**F**). The inset is the right-top cutout of “positive image” E and it marks where the spectra have been acquired.

After validation of the C-H nucleus visualization method, we demonstrate simultaneous fND detection. To do this we combine the KMCA luminescence pixel identification with a “negative image” created by C-H peak intensity mapping. For this experiment, we selected confluent cells with a lower fND uptake. First, Fig. 3A shows the “negative image” of non-labeled living MCF7 cells incubated with fNDs. Figure 3B then shows the same image including KMCA analysis to highlight the luminescent pixels. The image here consists of two stacked layers – the cell “negative image” in red merged with KMCA-detected NV-luminescent pixels (see Materials and Methods) demonstrating simultaneous label-free live imaging of cell nucleus and fNDs.

Further, we repeated these measurements on a fixed MCF7 sample preincubated with fNDs (Fig. 3C–F). These experiments demonstrate the possible use of our method for long-term experiments. In Fig. 3C, the whole C-H peak is mapped to create the cell image and the luminescent pixels are identified with KMCA. In comparison, Fig. 3D shows the nucleus visualization of the same data set by C-H “negative image” and Fig. 3E combines this image with KMCA detected fNDs. The chemical information from cells and the detected luminescent NV photons are combined in these images and it allows for colocalization of fNDs with the cell nucleus. We observe fNDs mostly in the perinuclear region, but some of them are located within the confocal detection volume of the cell nucleus and this is the case for both the living and the fixed MCF7 cells. The spectrum of a “chemical nuclear pixel” and a “luminescent nuclear pixel” for comparison are depicted in Fig. 3F. In both spectra, the C-H peak is clearly visible and the presence of the NV-luminescence is also apparent in the latter.

Detection of fNDs in the cell nucleus is an interesting feature of our study. The HPHT fNDs are generally believed not to enter the cell nucleus even though some penetration has been already observed for treated and much smaller (4 nm) detonation nanodiamonds^54^. The colocalization of NV and the nuclear signal can be explained in several ways. The first straightforward interpretation is that fNDs do enter the cell nucleus of MCF7. In several cases, inorganic and rather large nanoparticles have been observed in the cell nucleus^55^ even for MCF7^56^. However, even though the height of the cell nucleus of MCF7 is approximately 3 μm^57^ (substantially higher than the theoretical ~1 μm z-resolution of our confocal Raman system) there is a possibility that we detect the fND accumulation very close or even on the nuclear membrane. Moreover, the fNDs can be located inside the infoldings of the MCF7 nuclear membrane and these infoldings are smaller than the lateral resolution of the confocal microscope. To resolve this question, higher spatial resolution can be achieved by a combination of our Raman imaging method with super-resolution techniques such as stochastic methods of imaging or by stimulated emission depletion to detect fNDs^58^. The mechanism of fNDs penetration into the nucleus membrane is subject of our currently performed complex study and is not further discussed here.

**Figure 4:**
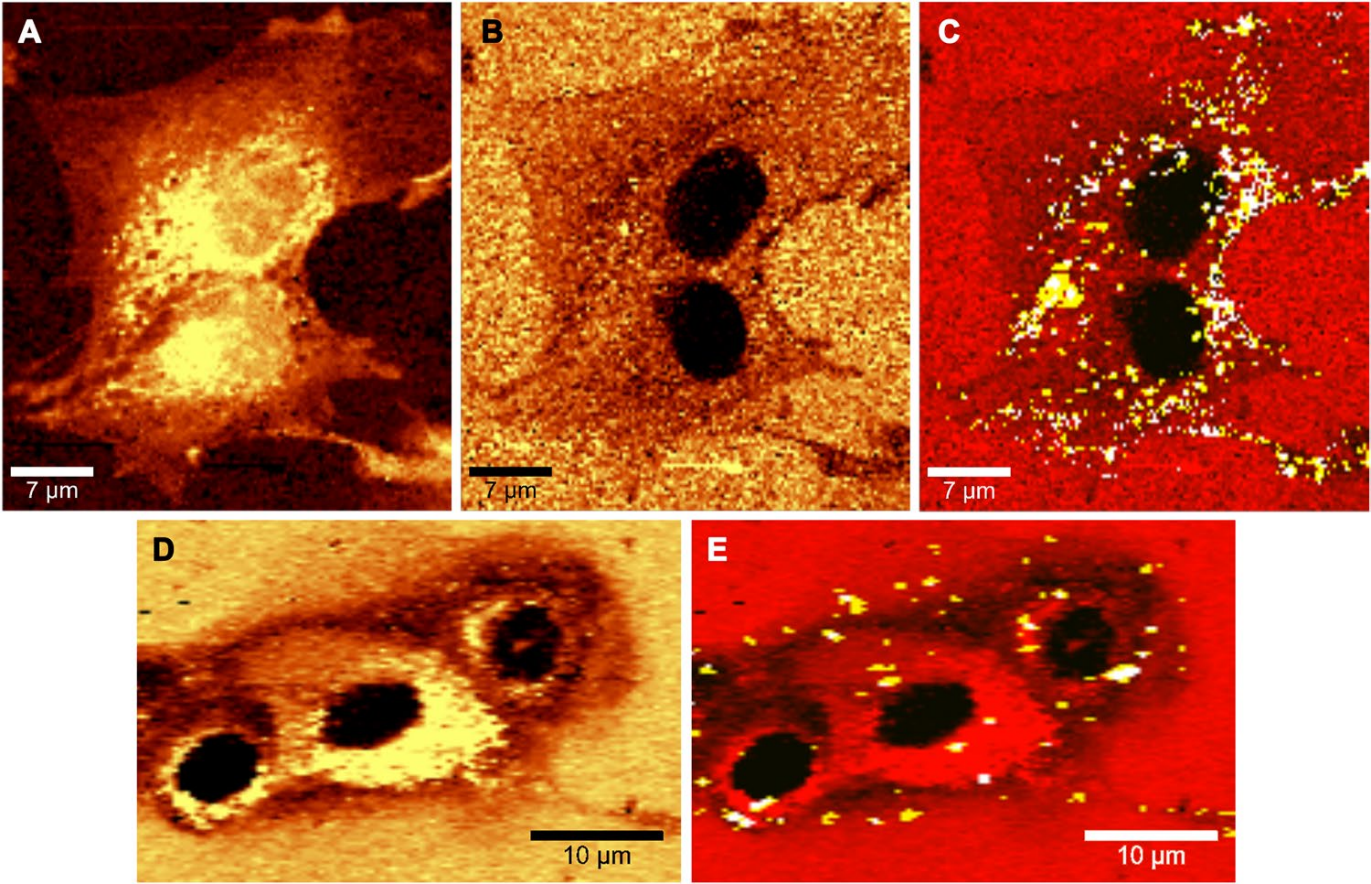
Simultaneous nucleus visualization with KMCA detected fNDs demonstrated on living DPSC cells (images A, B, C). Figure (**A**) shows the C-H image, (**B**) the “negative image” and (**C**) the merged results of “negative image” and KMCA-detected NDs. We show this technique also on 184A1 cells (images D and E) with the “negative image” in figure (**D**) and merged image in (**E**). For both cell types, we only show fND clusters with modulated C-H peak in the spectrum (yellow and white pixels) for clarity (images C and E).

Our technique to image the nucleus via Raman microscopy was demonstrated on both living and fixed MCF7 cancerous cell samples, but in theory, it could be applied to any kind of cell. Therefore, we further studied the capability of simultaneous fNDs detection and nucleus visualization on an additional two cell types. Experiments were carried out on human dental pulp stem cells (DPSC), which are the stem cells found in the teeth’s soft tissue^59^. These cells are very delicate and do not withstand long-term measurement under constant laser illumination and without proper conditions (37 °C and 5% CO_2_). However, since our technique requires only one confocal scan and low laser intensity (in comparison to CARS for example) to obtain all the information, we managed to image the living DPSC cells highlighting the cell nucleus and simultaneously detect the fND particles in standard laboratory conditions. Figure 4A shows the obtained C-H image, Fig. 4B the “negative image” of the same measurement, and finally Fig. 4C the merged image of DPSC in red together with yellow and white KMCA-detected NV-luminescent pixels proving wide-range applicability of our method. This time we clearly observe a higher density of fNDs around the edge of the nucleus, possibly localized at the nuclear membrane. However, because the height of the DPSC nucleus is lower than of the MCF7, we can only state that this detection is on the limit of the confocal axial resolution. In another set of measurements performed on mammalian breast cells (184A1) the nucleus could be again visualized clearly (Fig. 4D) and in Fig. 4E merged with the detected fNDs. Results on all three cell types show substantial nuclear contrast and high detection sensitivity.

## Conclusion

We have demonstrated a simple and efficient novel methodology for simultaneous visualization of luminescent fND probes and cell nucleus utilizing confocal PL/Raman imaging. The high contrast of the nucleus is obtained by modified Raman C-H peak imaging of the protein/lipid distribution in the cell. This method is robust and was verified on living and fixed cell samples and on three various cell types (MCF7, DPSC, and 184A1). To localize the fNDs, chemical information from cells and the luminescence from NV centers are acquired with a single scan and the fND image is reconstructed by *k*-means cluster analysis of the combined spectral data. Using this technique, we could directly distinguish internalized fNDs and demonstrate the colocalization of fNDs with the nucleus of MCF7 and DPSC within the diffraction-limited volume of the confocal microscope. We used small (5–50 nm) oxidized NV nanodiamonds. However, our method can be in principle employed to other quantum color centers such as silicon-vacancy, germanium-vacancy, and generally to any red- and near infrared-luminescent probes and allows to localize them with respect to the cell nuclei. The method is label-free and does not interfere with the cells, thus it can be applied to time-dependent measurements. It works also for fixed cell samples, which is favorable especially for long-term measurements. Combined PL/Raman imaging is specifically interesting for quantum sensing and imaging using NV centers in fNDs for which is the method aimed for. In addition, because fNDs can be functionalized and targeted to specific cell organelles, the presented method can be advantageously utilized as a simple, very effective, and precise tool for monitoring of fND-based drug delivery and simultaneous NV cellular sensing. Our method is also beneficial for the biocompatibility and cytotoxicity studies of fNDs in cells and specifically in the cell nuclei.

## Supplementary information

Supplementary Information.
