# Supplementary material for: Simultaneous label-free live imaging of cell nucleus and luminescent nanodiamonds
Source: Sci Rep. 2020 Jun 17;10:9791. doi: 10.1038/s41598-020-66593-7 (PMC7299945; doi:10.1038/s41598-020-66593-7)
Supplement: Supplementary file 1 — Supplementary Information. [file 41598_2020_66593_MOESM1_ESM.docx]

**Supplementary Information**

**Simultaneous label-free live imaging of cell nucleus and luminescent nanodiamonds**

*Michal Gulka^1,2,*^, Hamideh Salehi^3,*^, Béla Varga^3,4^, Elodie Middendorp^3^, Orsolya Páll^3^, Thierry Cloitre^4^, Frédéric J.G. Cuisinier^3^, Petr Cígler^5^, Miloš Nesládek^1,2^ & Csilla Gergely^4^*

1. Institute for Materials Research (IMO), Hasselt University, Wetenschapspark 1, B-3590 Diepenbeek, Belgium
2. Department of Biomedical Technology, Faculty of Biomedical Engineering, Czech Technical University in Prague, Sítná sq. 3105, 272 01, Kladno, Czech Republic
3. Laboratoire de Bioingénierie et Nanoscience (LBN), Université de Montpellier, Montpellier, France
4. Laboratoire Charles Coulomb (L2C), Université de Montpellier, CNRS, Montpellier, France
5. Institute of Organic Chemistry and Biochemistry of the CAS, Flemingovo nam. 2, 166 10 Prague 6, Czech Republic

* Both authors contributed equally.

**Raman imaging of cells:**

The commonly used spectral detection window for cell imaging using the 532 nm excitation wavelength is ranging roughly from 100 to 4000 cm^-1^, which corresponds to ~535 and ~675 nm respectively and to a grating central wavelength of ~600 - 610 nm (2000 - 2400 cm^‑1^). A typical measured cell spectrum with a 610 nm central wavelength is shown in Figure S1A and contains the “fingerprint region” (700 - 1700 cm^-1^), the C-H stretching mode (2800 - 3000 cm^-1^) used to create cell image and the Raman signal of water (3400 cm^-1^) from DPBS.

**Detection of fNDs in cells:**

We first image the internalized fluorescent nanodiamond (fND) particles (30 µg/ml, 1 hour) in live MCF7 cells keeping the common detection window with the central wavelength of the grating at 610 nm. To create the images we employ *k*-mean cluster analysis (KMCA) of the combined PL/Raman signal (see Main Text). The resulting average spectra of the KMCA-sorted clusters for the grating set at 610 nm are shown in Figure S1B. The obtained image in Figure S1C consists of two stacked layers. The first layer is the cell image created by mapping the intensity of the whole C-H stretching mode. On top, we highlight the pixels that contain the NV luminescence in their spectrum (from KMCA). However, we observed that using standard grating setting (commonly used to detect the “fingerprint region”) results in low recognition of fNDs for two main reasons. First, the main part of NV luminescence is undetected due to the chosen spectral range, which is cut-off at 658 nm, whereas NV emission is maximal around 700 nm. Secondly, the detected NV luminescence interferes with the C-H band and with the water Raman peak, consequently it is rather difficult to set the NV luminescence threshold properly to prevent false positive detection of fNDs inside the cells.

Therefore, we move the central wavelength of the grating to 680 nm, as there is no cell signal at around 700 nm (see the control spectra measured from non-incubated cells Figure S1D) and thus the detection of NV luminescence should be far more efficient. We then repeated the measurement on the same living cells at the same position. The resulting spectra of KMCA clusters can be seen in Figure S1E (same number of clusters as for Figure S1B). In this setting we still detect the C-H peak and also the majority of the NV luminescence, but we lose the characteristic “fingerprint region”. Thanks to detection over a larger spectral range of NV luminescence and straightforward distinction between luminescent and non-luminescent pixels, the sensitivity improves dramatically as can be seen from the image recorded in these conditions (Figure S1F).


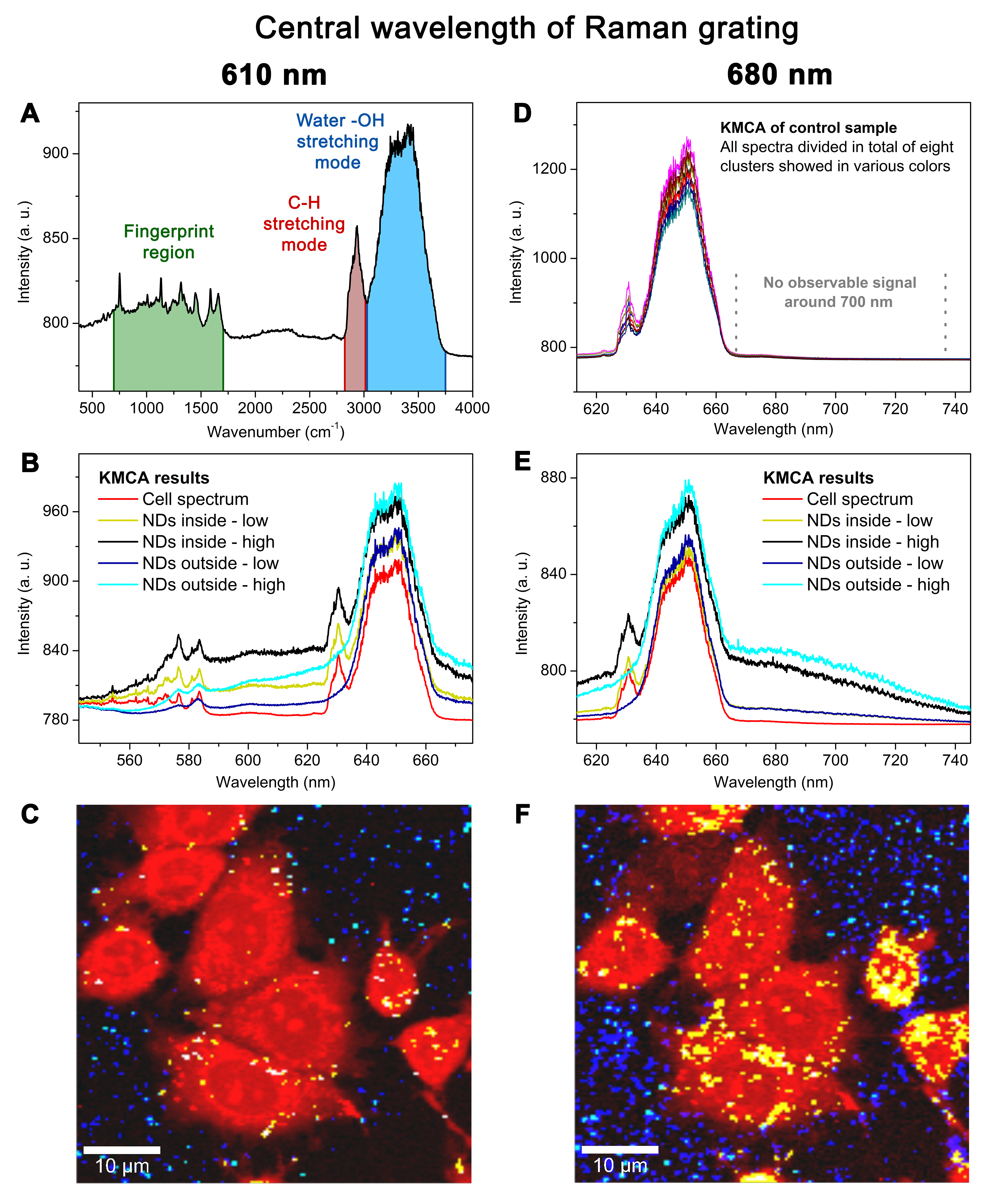


Figure S1: Comparison of PL/Raman imaging method demonstrated on non-fixed MCF7 cell line incubated with luminescent nanodiamonds in two consecutive measurements with grating positions set to 610 nm (left) and 680 nm (right). (A) The exemplary spectra obtained from non-incubated MCF7 with the central wavelength of grating set at 610 nm (common setting for Raman cell imaging) showing the “fingerprint region”, C-H peak at 2915 cm^-1^ from carbohydrates within the cell and H_2_O peak at 3400 cm^-1^ from the DPBS solution used for measurement. (B) The spectra obtained after ND incubation with the central wavelength of grating set at 610 nm showing average spectral shape of in total five clusters created by KMCA. Non-NV-luminescent cell signal cluster is shown in red and four NV clusters are shown in light blue, dark blue, yellow and white (see text). (C) Corresponding image created by merging the distribution map of Raman signal locked on C‑H peak (in red) with image of detected NDs (colors of luminescent pixels correspond to the color clusters in **B**, however black cluster is visualized as white in the image for clarity). (D) Spectra from all the pixels obtained by Raman imaging of the control non-ND-incubated MCF7 cell sample with the central wavelength of grating set at 680 nm. Lack of any signal at around 700 nm enables to lower the threshold for detection of NV luminescence and providing thus better sensitivity as demonstrated in **E**, **F** by re-measuring the same sample position as in **B**, **C**. (E) Averaged spectra of the obtained clusters for the repeated measurement and (F) the resulting image. With the central wavelength of grating set at 680 nm we obtain more than six times higher sensitivity for ND detection, however we lose the “fingerprint region” as a consequence.

**Comparison of different grating setting:**

The number of NDs detected is significantly increased compared to the previous image. With the grating set to 610 nm we identified in total 265 luminescent pixels inside the cells (with the C-H peak modulated on the spectrum) out of the total 10186 pixels that contained the C-H peak (cell pixels), which is 2.60% of luminescent cell pixels. For the grating set to 680 nm this ratio shifts to 1301 luminescent pixels out of 7927 cell pixels (16.41%), which is more than six times higher detection rate compared to the standard grating setting. Note that the difference in detection comes only from the improved identification of the luminescent pixels as no more NDs are taken up in between or during the two measurements (incubated cells are rinsed with DPBS and measured in DPBS without additional NDs presented). The lower number of the total cell pixels in second experiment is due to the detachment of one cell (left-top in the image) during the measurements. Since we get significantly higher ND detection when the NV luminescence does not interfere with the Raman signals, we carried out our further experiments with the grating central wavelength set to 680 nm.

**Utilization of C-H stretching mode for nucleus imaging:**

When looking closely at the resulting image of ND-incubated (30 µg/ml) living MCF7 cells (Figure S1F), it is noticeable that some NDs appear to be in the area where cell nucleus resides. However, as mentioned, the “fingerprint region” is not detected in the spectra and we could not resolve cell organelles and therefore prove or disprove whether the NDs are in the proximity of the cell nucleus. To solve this problem, we exploited the possibilities offered by C-H band visualization. Our technique allows in single scan simultaneous visualization of the nucleus of living unlabelled cells based on the shape of C-H vibrational spectra and detection of the NV-containing NDs compatible with ODMR measurements. Additionally, combination of the chemical information from the cells and the NV luminescence in the pixel spectrum is processed with KMCA. This allows to directly point out whether the NDs have been internalized in the cells and to obtain luminescence intensity maps by dividing the pixels according to the strength of the NV signal.
